# How an international research programme can contribute to improvements in the research environment: the perspective of doctoral students in sub-Saharan Africa

**DOI:** 10.12688/f1000research.144883.2

**Published:** 2024-06-24

**Authors:** Taghreed El Hajj, Neele Wiltgen Georgi, Susie Crossman, Nadia Tagoe, Imelda Bates

**Affiliations:** 1 Centre for Capacity Research, Liverpool School of Tropical Medicine, Liverpool, L3 5QA, UK; 2 Office of Grants and Research, Kwame Nkrumah University of Science and Technology, Kumasi, Ashanti Region, Ghana

**Keywords:** Research capacity strengthening, research environment, PhD programmes, research institutions, natural sciences research, sub-Saharan Africa

## Abstract

**Background:**

The Africa Capacity Building Initiative (ACBI) programme aimed to ‘strengthen the research and training capacity of higher education institutions and support the development of individual scientists in sub-Saharan Africa through UK-Africa research collaborations’ including by funding PhD studentships. We conducted research to understand students’ experiences and to see how consortia-based programmes such as ACBI and their own institutions can enhance PhD students’ research environment and progress.

**Methods:**

In-depth interviews with 35 ACBI-funded PhD students explored their perspectives about how their research and personal development benefitted from belonging to a research consortium. Questionnaires were used to corroborate interview findings.

**Results:**

Students recognised that membership of a research consortium provided many benefits compared to less well-resourced peers. By drawing on the programme and consortiums’ resources, they were often able to overcome some limitations in their own institution’s systems and facilities. Through their consortia they could access a wide range of international expertise and support from mentors and colleagues for their technical and psychosocial needs. Multiple consortia opportunities for engaging with the international scientific community and for networking, gave them confidence and motivation and enhanced their career prospects.

**Conclusion:**

Our study and its recommendations highlight how the breadth and diversity of resources available to PhD students through research consortia can be harnessed to facilitate students’ progress and to create a supportive and conducive research environment. It also underlines how, through a multi-level approach, consortia can contribute to longer-term improvements in institutional research environments for PhD students.

## Introduction

Strengthening research capacity in countries in the global south is a goal of many international research funders and development agencies because research can help to solve national problems and thereby drive socioeconomic development ( UNESCO, 2015). Sustainable funding to conduct research and to enhance institutions’ research environments is important for training, motivating and retaining researchers ( Colenbrander et al., 2015), yet most African research institutions continue to face serious funding shortfalls ( Teferra, 2013). Increasingly this is being addressed through collaborative arrangements for joint support from external partners and governments ( UKCDR, 2021). The UK spend on strengthening research capacity in low- and middle-income countries is vast. Between 2016-2021 the UK government and the Wellcome Trust alone spent £873M on dedicated research capacity strengthening schemes and £1.2B on projects that ‘add on’ capacity strengthening ( UKCDR, 2021). 80% of this spend was on African institutions, predominantly universities, and much of this was through research consortia.

Between 2012-2022 the UK Government’s Foreign, Commonwealth and Development Office (FCDO), in partnership with the Royal Society funded the Africa Capacity Building Initiative (ACBI), a pilot study to ‘strengthen the research and training capacity of higher education institutions and to support the development of individual scientists in sub-Saharan Africa through UK-Africa research collaborations’ ( Royal-Society, 2018). The ACBI programme focused on three research areas: i) water and sanitation, ii) renewable energy and iii) soil-related science and aimed to initiate lasting improvements in the research environment within the African host institutions. ACBI comprised ten research consortia, each involving one UK and three African institutions. Each ACBI-affiliated African institution hosted at least one PhD student: in total the ten ACBI consortia supported 38 PhD students based in 26 African institutions across 18 sub-Saharan African countries. Each consortium received funding for five years, including for PhD studentships which covered the students’ research expenses, travel and training and a contribution to equipment costs.

The Centre for Capacity Research (CCR) at the Liverpool School of Tropical Medicine was responsible for monitoring, evaluating and learning about capacity strengthening aspects of the ACBI programme and was not directly involved in any of the activities of the consortia or PhD students. As new learning emerged from the programme, this was disseminated to the ACBI management team, funders and consortia through regular meetings and presentations at programme conferences. In collaboration with all of the African and UK research leads, and with the PhD students themselves, CCR guided research in two phases to better understand and improve the capacity strengthening processes used within ACBI. In the first phase, and in partnership with the lead researchers in African institutions, we conducted qualitative baseline assessments of research capacity across eight ACBI-affiliated African institutions to identify existing capacity gaps and strengths, and to highlight examples of good practice and problem-solving strategies that could be shared within and beyond the programme. The assessments identified institutions’ strengths and gaps in postgraduate training and overall research environment against an evidence-informed benchmark ( Bates et al., 2014). A detailed account of these findings has been published elsewhere ( El Hajj et al., 2020). Whilst findings revealed significant differences in PhD programmes, institutions commonly experienced challenges with registration and induction, finances, communication, supervision, workload, infrastructure, research management and professional development and networking opportunities ( El Hajj et al., 2020; Pulford et al., 2020).

These findings clearly indicated a need to strengthen PhD training programmes but before making recommendations for improvement, it was important to explore the perspectives of the African PhD students themselves. This was the focus of the second phase of the research and of this manuscript. In particular we aimed to understand how to improve a) consortium-based PhD training and b) PhD programmes provided by institutions, based on the challenges identified and experiences of the cohort of ACBI PhD students, including creating a constructive and supportive environment where everyone’s contributions to research are valued.

## Methods

We used a mixed-methods approach ( Creswell & Clark, 2017) combining qualitative (interviews) and quantitative (questionnaire) data using in-depth interviews as the primary source of data complemented by quantitative self-administered questionnaires. This approach enabled us to validate findings from the interviews and to provide an idea of the weighting behind the students’ responses. The combined information could then be used to better understand which issues were common among the students. The PhD students were asked to consider the contributions made to their PhD experience by their institutions and their consortium at three levels – their individual development, the research infrastructure and support mechanisms, and their opportunities for interactions at the (inter) national level. To help ensure validity, the items included in the interviews and questionnaires were primarily based on findings from the baseline study ( El Hajj et al., 2020). These findings were then discussed with the African lead researchers to identify and prioritise issues that related to PhD programmes for inclusion in our data collection instruments. We also took account of published descriptions about the organisation of PhD programmes that were used to guide the baseline survey and informal feedback from the PhD students themselves about their experiences. Data collection instruments, consent forms and participant information sheets are available at https://doi.org/10.7910/DVN/DRKNED ( Centre for Capacity Research, 2024). We invited all 38 doctoral students directly supported by the ACBI programme to participate. We focused on the challenges that hindered their research progress, and the role ACBI played in addressing the challenges and in improving their institutions’ research environment.

### Data collection

*Interviews*

In-depth semi-structured interviews were conducted with 35 of the 38 ACBI-affiliated PhD students (92%): three students were absent at the time of the interviews or on maternity leave. All interviews, except one, were conducted in English and face-to-face between July 2018 and October 2019, either during site visits to ACBI-affiliated African research institutions or when students visited the UK. One interview was conducted online (in French) in October 2020 due to COVID-19 pandemic travel restrictions.

Interview questions covered topics that were previously identified as challenges in the baseline assessment such as student satisfaction with stipend; overall PhD progress; supervision; research outputs; challenges that might hinder or delay progress (at the personal, institutional and consortium levels); benefits from involvement in the ACBI programme; and suggestions for improvements. For equity purposes and to ensure all the students remained engaged in the research, they were all interviewed although data saturation was achieved after approximately three-quarters of the interviews had been completed (i.e., no new information emerged from the later interviews).

*Questionnaires*

Questionnaires were provided to all 38 students to be completed online. The majority were completed in May 2018, prior to any interviews being held. A few students were unable to complete the online form and were given the opportunity to fill them in either offline (via email) or in-person, prior to their face to face interview. All questionnaires were completed by December 2018. The overall response rate was 87% (33/38 students).

### Data quality assurance and analysis

Interviews were led by TEH supported by SC who observed and took notes during interviews; notes were then discussed and compared to clarify any discrepancies and to ensure and verify accuracy. All interviews, except one (at the student’s request), were digitally recorded after written and verbal consent was obtained. Recordings were transcribed, coded in NVivo software (under licence to Liverpool School of Tropical Medicine), and analysed using thematic analysis framework in Excel. Data coding and thematic analysis were carried out and discussed by two researchers (TEH and NWG) for verification and to ensure research rigour. Preliminary themes were presented and discussed with other research team members for their input and feedback. Quantitative data were analysed using the Statistical Package for the Social Sciences ( SPSS) software (under licence to Liverpool School of Tropical Medicine). For anonymity purposes, all quotes presented in this manuscript were given a unique number code to conceal the student’s identity.

### Ethics

Ethical approval for this study was granted by the Research Ethics Committee of the Liverpool School of Tropical Medicine (approval number: 17-061, approval granted 9 ^th^ July 2018). Informed verbal consent was obtained from each student after explaining the purpose of the study, the type of questions that would be asked, research procedure, voluntary participation, recording purposes, anonymity, confidentiality and privacy, risks, benefits and dissemination of findings. They were free to withdraw at any time.

## Results

*Characteristics of the PhD students*

Of the 35 PhD students interviewed, 60% (21) were males and 40% (14) were females. Of the 33 students who completed the questionnaire, 58% (19) were males and 42% (14) were females (mean age 37 years). The majority of students (61%; 20) were conducting their research in a renewable energy related field; this reflects that five of the ten ACBI consortia (22/38 students) had a focus on renewable energy. The remaining students were doing research in soil-related sciences (21%; 7) or water and sanitation (18%; 6). The majority (79%; 26) were in their second (13) or third year (13) of studies at the time they filled the questionnaire, and were mainly hosted at a “public university” (85%; 28/33). The rest were hosted either at a “research institution” (9%; 3/33) or at a “private university” (3%; 1/33). Only one student reported “other” for their type of institution. 79% (26/33) of students had “English” and 21% (7/33) had “French” as the main language of instruction at their host institution.

Themes that emerged from our analysis of the students’ interviews and questionnaire responses were divided into those that primarily focused on the infrastructure, facilities and research environment provided by students’ institutions and those that focused on the individual students’ supervision and personal development. Only one student did not want their interview recorded: instead running notes were taken during the interview. To avoid sharing personal health information or because of concerns about power dynamics, three students asked for the recording to be turned off temporarily and another three requested some information not to be disclosed. Overall the vast majority of interview data was available for analysis. We first describe the challenges experienced by the PhD students and then explain how many of these were mitigated by being part of a research consortium and, through the consortium, involved in the overarching ACBI programme.

## Institutions’ research infrastructure and facilities

Inadequate research infrastructure and facilities in students’ institutions occasionally hindered their PhD progress and also had implications for their motivation and longer-term retention.

“… *there are certain facilities that are not readily available to us and puts us at the downside when it comes to research because we may not be able to necessarily keep up with developed countries when it comes to cutting-edge research, mainly because of facilities. I do not want to attribute it to economic state of the country but unfortunately that plays a role too. I think we have a lot of technical know-how because most of the experts in the country are not trained here. They’re trained outside. But when they come back to help, they get frustrated because there are systems that don’t necessarily function and the facilities that you were trained with are not here*…”

    [007, PhD student, male]

*Institutional financial and procurement systems*

Inadequate or slow financial and procurement systems within students’ institutions impacted on the processing of their stipends and procurement of equipment and consumables. In some instances the consortia intervened to process stipend payments, procure equipment and pay for travel through a UK or African institution to avoid delays.

*Laboratory facilities*

80% of ACBI’s PhD students needed laboratory facilities for their research so challenges with these featured highly in interviews with students. They had difficulties accessing the laboratory itself or the equipment because it was being used by another student, researcher or laboratory technician, or else the laboratory was occupied by undergraduate students or equipment had access restrictions.

“ *Access to instruments. That’s the one thing I would say really becomes a challenge, because not only is it the limited resources, but also the number of students that get to use the same instruments. So, there’s quite a lot of waiting in-between.*”

    [034, PhD student, male]

More than half the students reported that their laboratories had dysfunctional equipment that occupied vital space but was not disposed of or repaired. Some students sought alternative ways of accessing the equipment they needed such as through commercial laboratories or other academic institutions, mostly in the UK.

*“You see, for our work we use a lot of instruments for the analysis, which are missing. And in most of the cases it’s the work of the university to supply that, to have it. So, without the instruments for your work, you can’t do much.* […] *Okay, I try to do whatever I can do with the limited facilities I have… like, when I was there* [in my home country] *what I was able to do was to prepare the materials so that once I am here* [in the UK] *, I can just characterise them using the facilities here.”*

    [035, PhD student, male]

Students who had to access facilities in external laboratories also experienced delays, for example, while waiting for health and safety training, or academic or technical supervision.

*“I stayed three months* [here at the UK institution] *but last time, it was worse to me … because they arranged the adviser to be here. When I came here, my advisor told me ‘It’s not my area’. So, I attended a master course on two courses, and I tried to work myself, to talk with my lecturer because they suggested the course can help me for my design.”*

    [028, PhD student, female]

Frequent power outages which delayed their research were reported by some students.

“ *Then the power, there’s no power. Sometimes I’m there for three weeks and there’s power maybe for five days out of those three weeks.*”

    [024, PhD student, female]

Although the ACBI programme provided for the purchase of research equipment and consumables to support PhD students’ research, a third of the students interviewed had encountered significant delays with procurement or sub-standard quality items.

“ *Another challenge for the synthesis is that you will need the chemicals and when you order the chemical it takes six months, even one year, or they are never delivered at all. And, you see, the challenge is, most of these chemicals, you don’t need just one chemical - you need one, three or five chemicals and you need to use them all at the same time.*”

    [035, PhD student, male]

*Access to literature*

Students often struggled to access journal articles and other e-resources through their institutional libraries so many found alternative sources. These included free, open access journals and platforms such as Sci-Hub and Google Scholar; requesting articles directly from authors; requesting articles through colleagues in institutions outside the country (including UK Principal Investigators); and by being granted library access through affiliation to a UK institution. Suggestions for improving access to literature included: provision of tutorials and support to students on how to retrieve literature and access resources (e.g., how to use search engines and literature databases); enhancing institutions’ subscription to scientific journals and databases; extending library opening times; improving access to computers, electricity and internet; and establishing collaborations with institutions with better access to literature.

*Questionnaire results*

The questionnaire findings from the 33 PhD students largely corroborated those from the interviews with the 35 PhD students from the same cohort. More than 80% of the students whose research was laboratory-based stated that new laboratory equipment purchased through ACBI was useful for the progress of their research work. More than half the students were “satisfied” or “very satisfied” with meeting rooms (66.6%), study area (60.6%), office space (54.5%) and teaching rooms/lecture halls (57.7%). Over half were also “very satisfied” with the power supply (60.6%), internet and IT/computer facilities (54.5%), library facilities (63.6%), and access to journals in their research field (54.5%). Barriers to accessing literature resources at students’ in-country institutions included lack of access to relevant databases/literature (64%), poor Wi-Fi/internet connection (45%), limited resources in general and in the repository (42%), and outdated resources in the library (36%). However, less than half (48%) were “satisfied/very satisfied” with their personal study space, access to relevant databases (45.4%), laboratory facilities (42%), and research software packages (39%). Lack of software packages caused particular frustration and delays for students doing computational research especially when combined with insufficient IT hardware, poor internet and interrupted power supply.

## PhD processes, supervision and progress monitoring

*Processes and supervision*

Students indicated that having a doctoral school (or equivalent) that had effective processes for admission, induction, progress monitoring and examinations, and which offered careers and financial advice, hardship funds and counselling and well-being services contributed to a positive experience. However, the students themselves had little or no involvement in framing policies and regulations at their institution relating to doctoral training programmes and student experiences.

The supervision experiences of the PhD students varied widely across the consortia; experiences that were supportive and encouraging were described as “motivating”.

“ *Usually, it’s a reinforcement by supervisors. They are integrated in our lives. I don’t know how to put it not to sound weird but … These guys are always encouraging you, especially if you’re actually working. I’ve worked under a number of people where if you go with the problem, no attention is paid to you or very little attention is paid to you. But these guys are involved. They’re with you the whole time. And they’re quite available as well, especially [Prof X] - he reinforces the fact that he believes in you. That’s the main motivation aside from the fact that you have family as well that are relying on you.*”

    [007, PhD student, male]

Students perceived good quality PhD supervision to be characterised by a having a supervisor with appropriate research expertise, and who was able to provide relevant technical and professional support. They also recognised that high-quality supervision also meant the supervisor provided timely responses and feedback to queries, was available for regular follow-up meetings, and provided clear and transparent communications and guidance. Four students mentioned that their lead supervisor lacked experience or knowledge in their research field. In a few cases, this led to the student having to repeat their work due to unreliable results leading to demotivation and lost time and resources.

“ *Prof has supervised a lot of students from various fields, but I think it would still be very good if there’s someone who knows exactly what you’re doing. I think it takes longer if you are finding that information all by yourself, but if you ask someone who is ahead of you, who has done something similar, you can tap from that person’s knowledge and move forward. I think that is a little bit not there.*”

    [019, PhD student, male]

Two students also reported inadequate support and unavailability of their main supervisor.

“ *The challenge is getting them. Sometimes you write, you remind them like five times, no one is responding. Of course, it discourages you.*”

    [014, PhD student, female]

*Student-supervisor relationships*

During the interviews students indicated that the relationship dynamics between students and supervisors are complex and hierarchical. Students perceived that they were very reliant on their supervisors for successful progression on the doctoral programme. This was because it was the supervisors who had to review their thesis, and provide feedback and technical advice, and who also facilitated the students’ future research, work opportunities, and professional and scientific networks. These dynamics were mentioned in almost all interviews regardless of the students’ satisfaction level with their supervisors and their overall supervision experience.

Some students acknowledged and accepted that “respect” for the supervisor’s position, knowledge, expertise and even age, was the basis of a “professional relationship”. A few students felt frustrated and anxious with the imbalance in student-supervisor power relations and expressed challenges in managing differences of opinion with their main supervisor, to the extent that most of the dissatisfied students were only willing to provide “off-the-record” information.

“ *Clear guidelines guarding both students and supervisor must be made and communicated to both. Until you get into trouble, you sometimes do not know some guidelines exists.*”

    [011, PhD student, male]

*Joint supervision*

Under the ACBI scheme, the Royal Society strongly encouraged implementing a shared supervision between the UK and African institutions. In the vast majority of cases, this proved to be a successful approach that PhD students found valuable and constructive as it gave them access to diverse expertise, knowledge, resources and discussions and enriched the scientific input to their research work. However, for a few students this resulted in an overwhelming number of supervisors. For instance, four students reported having between four and six supervisors because their own institution allocated two to three local supervisors and they were also assigned external supervisors from their ACBI consortium. Whilst students acknowledged the value of having various supervisors with different areas of expertise to add richness to their research, they also stated that managing timely feedback and discrepancies in opinions and reaching a consensus on ways to move forward could be challenging and delay progress.

“ *I have 6 supervisors … consulting with so many supervisors with different opinions gets me confused and sometimes delays my work progress* …”

    [003, PhD student, female]

*Monitoring PhD progress*

All students interviewed had formal or informal progress monitoring by their own institution (i.e., by their supervisor, department or graduate/doctoral school) or by their ACBI consortium. These included filling out progress forms (each semester or annually) which had to be signed by their supervisor and submitted to the graduate school at the university. A couple of students thought these processes not effective. Other mechanisms included meetings (weekly, monthly, ad hoc) with their primary supervisor or presentations at departmental and consortium meetings.

“ *Every month … the last Friday of every month, I’m supposed to meet Prof. It doesn’t happen all the time because sometimes he’ll get busy and all that. I go to him, I tell him what I have done, what I’m doing – for example, some occasional informal outputs: I’ve made a couple of reports—presentations and groups of lectures* …”

    [019, PhD student, male]

Students found presentations to groups to be useful because they stimulated debates and discussions which were learning opportunities not only for the student but also for other researchers and fellow students.

*“Yes, quarterly almost* [student present their work progress to supervisors and department]. *Then, as a consortium, every time we meet for consortium meeting, we have a presentation which show the way we progress our research. Also, apart from a consortium meeting, we have summer conferences where we have to give our presentation and then you have to let your supervisors know. It is also a way of monitoring your progress.”*

    [026, PhD student, male]

Some students were required by their institutions to provide regular written reports in order to obtain their stipend, or to present their progress to formal supervision panels convened by their institution or their ACBI consortium.

“… *Every six months we submit a six-month progress report to the faculty. We have to show the results, give the aim, objectives of the results.*”

    [030, PhD student, female]

“ … *We have a committee that is organised by department where you have your supervisor, the student and two outsiders from your field. We do have a meeting every six months. [….] Yes, without the general meeting, we have a progress report every month, myself and my supervisors. Then the general one is every six months. That contains other groups beside the supervisors and students.*”

    [006, PhD student, male]

For two students their consortium tracked their progress by monitoring their data inputs into a database:

“[The UK PI] *created a page* [at UK University X] *where almost every data you have finished, you submit it to that page. Yes, a format yes where almost all the data we are having for most of us it is still in a raw form but what we have been able to type and send, you send it to that page … No set monitoring system in place with the supervisor.”*

    [011, PhD student, female]

The majority of students were confident that any delays in progress would first be discussed with their supervisor(s), then with the UK consortium lead before any further action was taken at a higher level (either by their institution or the ACBI programme).

*Questionnaire results*

Although 58% of students reported being “satisfied” or “very satisfied” with communication by their graduate school, the majority reported that they did not receive any financial hardship (88%) or career advice (61%), or any counselling or well-being services (55%). Only 12% of students reported “ever participating in developing/updating post-graduate related policy and strategies at their department/institution”.

The vast majority of students (94%) had had “good” or “satisfactory” quality of PhD supervision and 82% described their relationship with their main supervisor as “good” or “very good”. Most students (79%) considered their lead supervisor to be an expert in their subject area and 67% “always” received enough support from their main supervisor(s). 64% “always” received timely feedback from their supervisor(s) with a turnaround time of two days to one week. Over half the students (52%) met with their supervisors at least once a month and 33% met with them at least once a week. 67% of the students were aware of the PhD progress monitoring processes/guidelines at their institution but only a few (21%) were aware of the institutional procedures and actions taken if progress was unsatisfactory.

### Personal and professional development

*Students’ enhanced knowledge and skills*

Almost all the students were enthusiastic about their participation in ACBI recognising it as a great opportunity and a privilege. Many of the students interviewed were aware that professional development opportunities provided by their host institutions for PhD students were very limited due to the lack of funding. Almost all students considered themselves to be privileged because through their consortia and the ACBI programme they could access a wide range of technical and research skills training opportunities. They recognised that these opportunities would likely be unattainable and unaffordable for their colleagues who were self-funded, non-ACBI PhD students.

“ *My research skills have been built in a lot of ways, I have learnt new research methodologies, I have learnt new statistical approaches, I have learnt new laboratory techniques.*”

    [001, PhD student, female]

Students reported a significant increase in self-confidence, knowledge, and skills from their participation in ACBI. For instance, many PhD students felt equipped to mentor and train their peers, masters students and undergraduates within their home institutions, and felt empowered to progress into fellowship programmes and lectureship positions after graduation.

Around a third of students were involved in teaching at their institution. Despite the additional workload they recognised that this improved their knowledge and skills, and positively contributed to their research work and career development.

“ *I cannot complain because in our consortium we do most of the time visit other institution partner. Also, sometimes, we do organize - every year we have this consortium meeting. When we arrive, sometimes we present and organize some workshop with students in the department. That’s it. Sometimes we do have training, a lot of activities. In our case, I will say, so far, we got my team, I can say that the research capacity is going on well.*”

    [020, PhD student, male]

“ *I learnt a lot from sharing my knowledge with other students although this sometimes puts pressure during peak teaching times and during my fieldwork.*”

    [002, PhD student, female]

A couple of students even suggested making “teaching undergraduates” part of the requirements for PhD students under the ACBI programme.

“ *Teaching experience has pushed me to mature from the stage of constantly being taught to a point where I can supervise undergraduate projects.*”

    [007, PhD student, male]

*“I’ve benefited greatly with the interaction with* [field technician X] *I think…Also seeing somebody’s life improve because of this project has been very amazing. To watch* [the field technician’s] *confidence grow and just his life has just got better.* [Principal Investigator X] *often says that this that’s come out of this project is very ready to be celebrated because he cares so deeply about the project, he tried to understand it and everything.”*

    [038, PhD student, female]

Some UK PIs made efforts to engage students in other projects beyond the ACBI programme allowing students to learn how to manage projects, encouraging them and opening new opportunities for them.

“ *One major benefit is networking with other PhD students and African researchers and researchers in the UK … I hope our relationship continues beyond the programme, and I think it will* …”

    [003, PhD student, female]

Francophone students reported improved English language skills, especially for those who spent a few months in the UK and interacted with researchers and academics in English.

“… *Secondly, I improved my English. I learned English in secondary school but because I never communicate and I was not a part of an English programme, it is not something that matters for me to improve or to do any effort to communicate in English. From this program I’ve seen that we have to learn.*”

    [010, PhD student, female]

*Mentorship and peer support*

Most students had received mentorship, though only two described this as ‘formal’. Mentorship was provided by senior colleagues at their own institution (e.g., lecturers, postdoctoral researchers, laboratory technicians) or by ACBI-affiliated research fellows or PhD students. Mentors facilitated exchange of scientific ideas and involvement of students in training and consultations. They provided technical and moral support and encouragement. They gave advice on research opportunities and future career direction, reviewed documents and provided feedback on students’ scientific writing and overall research work.

*“I’ve got some informal mentorship, especially that we’ve already got to the writing* [stage] *. At* [X] *University, we’ve got two guys who used to deliver scientific writing sometimes …. When you write, you submit, and they can check it for you and to give a few feedback … It has been very helpful for me because I used to have it as a pre-processing of my writing before I submit to my school supervisor."*

     [026, PhD student, male]

*“*[They have a] *mentor in a fellowship program that I was involved in …. So, she was my former mentor, because it’s a mentorship program, and I have since graduated from that program, but she has continued mentoring me morally, and also even […] she is one of those I can have a document and ask her to proof-read for me, or to go through it.”*

     [001, PhD student, female]

Students valued the academic and professional support, and advice and problem-solving offered through their consortium, and from regional and international experts.

*“They* [African and UK researchers in the consortium] *bring in a very good level of expertise, so I have benefited through their expertise; and even the methods that I’m using through my study are quite new to me, and all that has been facilitated through this consortium.”*

    [001, PhD student, female]

Two students stated that they would have benefited from having an “independent mentor” (outside their department and/or consortium) whom they could confide in and reach out to when faced with difficulties from their supervisor(s).

A quarter of the students reported that positive interactions (e.g., sharing research ideas, peer reviews, support with methods and data analysis) with other students, colleagues and senior researchers contributed to a positive working and learning environment. However, a few students felt they lacked a supportive environment mainly due to a competitive research environment at their institutions. For instance, students indicated that some lead researchers did not encourage the advancement of PhD students and early-career researchers, particularly if they felt exposed or insecure about their own academic performance.

“ *Spirit of collaboration needs to be enhanced and unhealthy competition among lecturers, and lecturers and students should be subdued.*”

    [011, PhD student, male]

*“… maybe they* [supervisors or lead researchers] *feel threatened. They fear that the student might do better than the supervisor.”*

    [030, PhD student, female]

*Networking*

Students indicated that opportunities for networking, and for establishing long-term professional partnerships and academic collaborations (such as shared publications, grant writing, knowledge sharing) were some of the most valuable benefits of belonging to ACBI. These opportunities included South-North and South-South exchange visits, scientific meetings and conferences, and training workshops. These enabled students to interact with a wide range of researchers, experts and scientists from within and outside their research field and to participate in joint research activities within and beyond their consortium.

“… *it’s giving me so much exposure just in terms of the conferences that I’ve been able to attend and the links that I’ve been able to establish. I know and communicate with people almost from every corner of the world just because of this one project.*”

    [024, PhD student, female]

These activities allowed the students to strengthen their communication skills and confidence through networking and presenting research findings.

“… *presenting at the meetings and conferences, it gives you the chance to speak. You get more confidence in yourself in presenting and all [the networking].*”

    [030, PhD student, female]

Importantly this networking built a sense of solidarity among the whole cohort of African ACBI PhD students and enabled them to create African communities beyond their own consortium on specific research topics using WhatsApp.

“ *The collaboration with the south-south has been really beneficial … We’ve been able to collaborate with each other, learn from each other and become inclusive with the research that we are doing, and even it’s kind of built this solidarity amongst the [African] nations … even amongst the students, apart from times when we meet during the consortia meeting, when we go back to our different countries, we are able to communicate with each other and talk about our projects and update on our progress.*”

    [003, PhD student, female]

All students interviewed reported communicating with other PhD students “within their consortium” and around half reported communicating with PhD students “across other research consortia within ACBI”. These interactions were mostly perceived as beneficial for providing peer-to-peer support such as sharing and discussing the challenges they faced; discussing solutions and good practices; learning about new/innovative research methods and techniques; reviewing each other’s work (peer-review); and advising on ways to improve research work.

*“Our group, I’ll say, it’s a rainbow … Different colours all matched together. That’s what I love about it … Perfected by its colours - so like everybody is from their* [own] *discipline, but usually, even in the meetings you see that there’s no difference between* [us] *despite the* [specialised work] *that they are doing …* [We communicate] *a lot … WhatsApp, emails … I think we have moved from being colleagues to being siblings. Because I remember one time, I was having a problem with my* [X] *experiments, so I made a WhatsApp call, so we ended up having a video call training and it was three hours long… That’s how we are usually …”*

    [008, PhD student, female]

In rare cases, competition between students within the same consortium hindered collaboration and communication between the students and created a feeling of isolation and discomfort at the beginning of the programme. However, as soon as their interaction and communication with each other and with students from other consortia increased, they started to open up, share information and help each other. This eventually created more solidarity, understanding and support for each other.

*Questionnaire results*

Questionnaire data supported the interview findings, showing that the vast majority of students (88%) felt “advantaged compared to other PhD candidates at their department/school”. More than half (58%) of students reported being “satisfied” or “very satisfied” with the access to personal development and skills training opportunities; and 70% reported being “satisfied” or “very satisfied” with their opportunities to participate in conferences, workshops and competitions. More than half the students (61%) had a mentor other than their supervisor (e.g., post-doctoral/senior researcher) and 67% received support from other PhD students. Students utilised their consortia for “participating in capacity building activities organised by the consortia” (76%); “knowledge exchange” (61%); “building network for career related reasons” (55%); and for visits to UK partner institutions (52%).

### How consortia membership benefitted PhD students

Our findings indicate that students had identified several hinderances to progressing their PhD studies that were beyond their control and which were mostly related to inadequacies in their institutions’ research infrastructure and systems ( Box 1). Many of these were recognised by consortium leaders and the ACBI management team and mitigated for the student through their own consortium within the overall ACBI programme. In particular, ACBI invested heavily in providing equipment for the research consortia and to support PhD student’s projects. This meant that some students no longer needed to travel to other laboratories to conduct their experiments. Some departments even used their own funds to refurbish their laboratories to accommodate the new equipment and instrumentation purchased through ACBI. The new laboratory equipment, computers and software also benefited other researchers, students, and research support staff such as laboratory technicians.

Box 1. **Hindrances to PhD students’ progress and mitigations provided through their ACBI consortium or programme.**

**Table:** 

Hindrances to PhD progress	Mitigations provided by students’ consortium or ACBI programme
***Research support systems***	
▪ Delays in financing which affected equipment purchase and stipends ▪ Slow procurement processes ▪ Unreliable power supply ▪ Lack of laptop/computer * ▪ Insufficient time allowances for maternity leave ▪ Lack of access to up-to-date resources (e.g., books, manuals, research articles) ▪ Lack of quiet study space	▪ Laptop provided for each student ▪ Improved access to scientific literature e.g., through granting access to UK university libraries during exchange visits, and consortia members providing articles on request ▪ Advocated for better study and learning space that positively impacted on PhD studies ▪ Research consortia processed funds/stipends and paid for travel which avoided payment interruptions and delays ▪ Improved financial administration and procurement (e.g., faster turnaround of funding claims) ▪ Extended PhD timelines to account for maternity and sick leave
***Laboratory facilities***	
▪ Inadequate laboratory and field equipment including licences for computational software ▪ Lack of, or sub-standard, laboratory consumables and reagents ▪ Restricted access to laboratories and/or lack of laboratory availability to conduct research	▪ Purchased quality consumables and new equipment (e.g., GPS, cameras, projectors, laptops, software, high-performance computers) that benefited the students and their institution ▪ Granted PhD students access to sophisticated laboratory equipment unavailable in their own institutions, through exchange visits to other institutions in their consortium
***PhD processes***	
▪ Lack of the technical training to do research work * ▪ Inadequate PhD supervision ▪ Security issues hindered fieldwork ▪ Lack of professional development opportunities	▪ Provided technical and generic training (including through exchange visits) ▪ Provided PhD students with opportunities to present at scientific conferences and meetings – which enhanced their self-confidence and communication skills, and where they learnt about different research cultures/systems ▪ Improved English language skills and networking with Francophone institutions, among Francophone students ▪ Consortia provided academic and professional support, advice and guidance from a range of world-class experts and supervisors (including assigning supervisory panels and formal monitoring of progress) ▪ Networking and collaboration between PhD students and their supervisors which fostered high quality research and research outputs

* *Note that laptops and training were provided but may not have been in place for all students at the time of this study.*

*“… we’ve had funds to do some previously, say, impossible things. We’ve bought so much equipment, some of them we used to maybe dream about them. We’ve finally had the funds to buy this equipment. We’re doing some ground-breaking research.*”

     [024, PhD student, female]

33% of the students observed changes in the infrastructure and learning environment that they attributed to the ACBI programme, and which positively impacted on their PhD programme and experience.

## Discussion

The overall aim of this study was to identify actions that were then used to improve the ACBI programme within its lifetime. More broadly we also aimed to explore how institutions and consortia-based programmes can help to create a constructive environment that supports PhD students’ progress and wellbeing. From data focused on the perspective of 35 African PhD students we have formulated recommendations that will be useful for institutions to improve their own doctoral programmes and for designing other large, multi-national, consortia-based research programmes. Our complementary findings about PhD programmes from the institutional perceptive have been published previously ( El Hajj et al., 2020).

Creating a supportive research environment and a sense of belonging to a department is an important part of strengthening research capacity including for the progress, interest, empowerment and retention of PhD students ( Langhaug et al., 2020; Pyhältö et al., 2009; Stubb et al., 2011; van Rooij et al., 2021). A supportive research environment is one which is inclusive and values everyone’s contributions to research; it encompasses the behaviours, values, expectations, attitudes and norms of research systems ( Moran et al., 2020; Royal-Society, 2021; UK Government; Department for Business, 2021).

Our findings about the students’ perspectives fell into three categories: factors affecting the infrastructure and systems of institutions hosting the students; the PhD processes and student supervision; and students’ professional development, though – since our participants were PhD students – most of the findings concerned the last two categories.

Previous studies of institutional research capacity ( Pulford et al., 2020) and PhD programmes in low- and middle-income countries have shown that access to adequate research facilities and appropriate infrastructure is a major enabler for sustainable research capacity ( Ogundahunsi et al., 2015; Pilowsky et al., 2016) and for timely completion of PhD programmes ( Pitchforth et al., 2012). Similar to our study findings, a large Africa-based programme that provide strategic support for PhD student cohorts was effective for creating opportunities for networking and research collaboration as well as enhancing research knowledge and skills ( Adedokun et al., 2014). This programme’s curriculum was intentionally designed to facilitate critical reflection and as a result the students reported shifts in their sense of self, worldviews, and the definition, construction and evaluation of knowledge ( Ruhweza Katahoire et al., 2023).

Consortia were able to circumvent several weaknesses in institutions’ infrastructure and systems thereby reducing hindrances to students’ progress. For example, they were able to arrange timely procurement of good quality equipment and consumables. Although ACBI did not fund building or infrastructure costs, the programme was able to advocate for improvements in internet access and power supplies for students, and for their need to have a quiet space in which to study and write.

ACBI’s focus on natural science research meant that accessing up-to-date research equipment (laboratory or computational) was commonly mentioned as essential to their successful progress. Through their consortium, many students were able to use world-class equipment for their research either because this was purchased for their own institutions or because they had funds to travel to other institutions within their consortium where such equipment – and training on its use – was available. Despite the support from ACBI, students still faced difficulties with their institutions’ slow procurement processes and unavailable or sub-standard consumables and equipment, and even when the equipment was procured internationally, there were often delays at customs. Issues with procurement delays and poor-quality equipment and consumables, despite funding from external programmes, have been found in previous studies ( Pulford et al., 2020) suggesting that this is a widespread and intractable problem that is a substantial barrier to effective research, that would be a good target for a focused joint effort by institutions and their collaborators.

An innovative aspect of the ACBI programme was the formalisation of arrangements to transfer ownership of the equipment to the African partner institutions along with an agreed maintenance and sustainability plan. Access to scientific literature and up-to-date resources is essential for PhD students to learn about the latest discoveries, innovations, problems and methods in their research field ( Colenbrander et al., 2015; Davis & Walters, 2011). Through their consortium, PhD students were able to have much better access to scientific journals than many of their own institutions were able to provide. Some students were even given access to the libraries of their UK partner institutions. For others, members of institutions within the consortium partnership were able to provide students with the articles they needed.

Previous studies have indicated that supervision quality, good communication and the relationship between the student and supervisor are vital for students’ motivation and progress ( Ali et al., 2017; Cotterall, 2013; Ives & Rowley, 2005; Khozaei et al., 2015; Lee, 2008; Leijen et al., 2016; van Rooij et al., 2021; Welde & Laursen, 2008; Young et al., 2019). Positive supervision experiences motivate students, increase their self-confidence and create a constructive drive for their work progress. A negative supervision experience can lead to frustration, delays and demotivation. The PhD students in the ACBI programme benefited by having supervisors from within their own institution and from their consortium. This gave them access to a broad range of expertise in their subject area and more personal support than would have been possible for students who were not part of a large programme. The vast majority of students were satisfied with their PhD supervision and their relationship with their supervisors.

A few students were dissatisfied with the quality of their supervision and attributed this to their supervisor having insufficient knowledge of the research topic, being unavailable or unable to provide clear guidance or timely feedback, and tensions in the supervisor-student relationship. Reasons for poor quality PhD supervision highlighted in previous studies ( Lee, 2008; Leijen et al., 2016; Young et al., 2019) include supervisors’ overwhelming teaching and administrative workloads ( Lee, 2008; Pulford et al., 2020), their styles and approaches to supervision ( Lee, 2008), and lack of accountability and oversight of PhD supervision ( Colenbrander et al., 2015). Students’ own characteristics, such as their research skills ( Ali et al., 2017; Khozaei et al., 2015), knowledge and experience, communication and management skills, and their ability to write academically ( Khozaei et al., 2015) and to critically reflect, also contribute to PhD supervision quality and progress ( Lee, 2008).

Despite the high levels of satisfaction with their supervision, the ACBI PhD students made several suggestions about how their supervision could be further improved. These included establishing clear roles and responsibilities for students and their supervisors, formalised tracking of progress, and training for supervisors in communication, leadership, management and supervisory skills. Such suggestions have been made previously along with clearly defined standards for programme admission and supervision of doctoral students, improving students’ critical thinking abilities, and enhancing supervisors’ skills so they can develop relationships in which students feel motivated, inspired and cared for ( Ali et al., 2017; Duke & Denicolo, 2017; Lee, 2008).

Development of confidence and interpersonal skills is highly valued by doctoral students ( Lindén et al., 2013) and is important preparation for their future whether it is within or outside academia ( Duke & Denicolo, 2017; Lindén et al., 2013), but is often an overlooked component of PhD programmes ( Lee, 2008). This was not the case in ACBI which provided multiple opportunities for students’ personal and professional development covering technical and soft skills such as academic writing and presentation skills. These opportunities included technical training, language skills (for French-speaking students), exchange visits, conference attendance and scientific discussions with world-class experts in their field.

Through ACBI, students had a large pool of colleagues and mentors they could turn to for academic and personal support during different aspects and stages of their programme. This support took various forms including reviews of data analyses and draft publications which improved their quality, discussions to help guide students’ careers, and personal and psychosocial support. Students recognised that their acquisition of new knowledge and skills, their expanded networks and collaborations, and all-round support provided by their consortium for their research and personal needs, had boosted their career prospects, self-confidence and motivation.

### Suggestions for how institutions and consortia can enhance the research environment for PhD students

By working together and taking a multi-level approach to capacity strengthening, institutions and research consortia programmes can attain consortium goals as well as systemic and sustainable improvements in institutions’ PhD programmes. Below we have summarised the suggestions proposed by the African PhD students in this study about how their institutions and consortia can contribute to a conducive research environment, and how they can mitigate barriers to progress faced by the students ( Box 2). In addition to promoting sustainable PhD training benefits, adoption of these suggestions would help to provide the kind of environment that the PhD graduates can thrive in beyond the consortium period.

Box 2. **Suggestions for institutions and research consortia: how to contribute to a supportive research environment from the perspective of African PhD students.**

**Table:** 

**Research facilities** • Provide access to quality consumables and state-of-the-art equipment, and reliable power supply • Streamline and speed-up procurement processes for research equipment and supplies • Provide access to quiet study space and to research journals • Advocate for adequate IT capability **Supervision and mentoring** • Set out the role and responsibilities of supervisors and students, including reciprocal timely feedback • Ensure supervisors have expertise and research skills to match the students’ research topic • Ensure supervisors have adequate time to provide quality supervision and that they are held to clear and appropriate standards • Provide training and ongoing support for supervisors including in technical, communication, supervisory and management skills • Establish formal induction and progress monitoring processes for students • Ensure students have a manageable number of supervisors • Provide students with access to mentors (formal or informal) to provide additional research expertise, psychosocial support and careers and personal guidance **Personal development** • Provide training in technical skills, critical thinking, and in research management and leadership; tailor this to meet students’ needs and career aspirations • Facilitate exchange visits for technical training and exposure to different research environments and systems • Foster supportive and inclusive collaborations among the PhD cohort and with other researchers and research support staff within and beyond their institution and consortium • Provide opportunities for (inter) national networking with peers and research topic experts, and facilitate collaborations to enhance career and research opportunities

Our study highlighted areas for further research including:

• How can consortia facilitate more efficient procurement systems in partner institutions, sustainably and in a way that does not undermine the institution’s own systems?
• How to foster a sense of belonging to a department among PhD students?
• How does the transfer of project equipment ownership to partner institutions, along with agreed maintenance and sustainability plans, impact institutions’ research performance (e.g., enhanced research opportunities and potential for income generation)?
• How can inconsistencies in PhD supervision be minimised to ensure that all PhD students receive high quality academic support?

Some of these research questions concerning the challenges faced by PhD students cannot be directly addressed by consortia because they depend on changes in institutions’ broader research environment and need long-term efforts and investments. Nevertheless, externally-funded research projects need to be cognisant of these and have a role to play in catalysing improvements. For example projects can:

• Involve institutional leaders/stakeholders in their capacity strengthening plans and share emerging findings with them
• Promote institutional investments (possibly with project co-funding) to address systemic challenges for PhD training (e.g., providing and refurbishing space for new equipment or study space, and for improved internet)
• Facilitate PhD-relevant policy development and revisions, and the involvement of PhD students in these processes
• Promote counselling and well-being services for PhD students
• Facilitate strategic institutional collaborations with long-term potential beyond the project’s lifetime (e.g., through shared use of laboratory equipment)
• Set up pilot institutionally-owned projects to tackle systemic challenges (e.g., concerning financial and procurement procedures) which are scalable beyond the project lifetime

In using these and other actions to enhance the research environment it is important for international consortia such as ACBI to collaborate closely with their partner institutions. They need to actively discover and build on the strengths of the institutions and to avoid undermining their systems, and make sure that their efforts are complementary to the vision, strategy and needs of partner institutions.

### Strengths and limitations

Our study had several strengths and limitations. Our participants were a large cohort of contemporaneous African PhD students who were part of the ACBI natural sciences research programme. The size of the cohort, the balanced gender mix, their diverse institutions and multiple countries meant that the students had many different perspectives which provided broad and deep data for the study. The students all belonged to the same programme (ACBI). This reduced confounders due to differences in management processes and access to resources that would have been present if they had all been affiliated with different programmes. However, as all the students were doing natural sciences research, there was a strong emphasis in this study on laboratory facilities; findings relating to these aspects may therefore not apply in the context of non-laboratory research. Despite ACBI’s natural sciences focus, the variety in types of studies and research topics ( Royal-Society, 2018) means that the findings could be applicable to science PhD students across sub-Saharan Africa and in other low- and middle-income countries. However we are aware that the ACBI students were relatively well-resourced and had access to more resources and opportunities for mobility and supervision compared to students who were not part of a an international programme. We were aware that students may have felt constrained in providing perspectives that may be construed as critical of their institutions or supervisors. We mitigated this by ensuring that all responses were anonymised, by assuring students of complete confidentiality and advising them they were able to withdraw their comments or participation at any time.

## Conclusion

Our study has highlighted several ways in which research consortia can contribute to conducive environments for PhD students, and how they can make the students’ experiences positive and fulfilling. Unlike more senior researchers, PhD students’ experiences are critically linked to their relationship with, and quality of support from, supervisors and mentors, and this was reflected in their interviews and in our recommendations. Since they were natural science students, the quality and availability of equipment and consumables was also a common focus of their recommendations. The networking and collaboration opportunities provided by consortia were highly valued by the PhD students with many intangible benefits such as confidence-building and enhanced career prospects. Our study findings provide valuable insights into how consortia can complement and contribute to improvements in the PhD programmes and facilities of their member institutions and promote a conducive research environment in which the students can feel supported and flourish.

## Data Availability

Transcriptions of interviews have not been made available as a dataset because they cannot be de-identified without compromising anonymity and the project’s ethical approval conditions. These limit the access to raw, unprocessed data strictly to the research team to safeguard the privacy and confidentiality of participants. Despite these constraints, the authors are open to discussing specific requests for further information (via the corresponding author) about the contents of the transcripts while strictly adhering to ethical obligations. Harvard Database: Extended Data - How research consortia can contribute to improvements in PhD students’ research environment and progress in sub-Saharan African countries, https://doi.org/10.7910/DVN/DRKNED ( Centre for Capacity Research, 2024). This project contains the following extended data:
• ACBI Online Survey_PhD candidates 2018_Unrestricted_FINAL.docx • Interview Guide PhD Students-Phase 2-FINAL.docx • Participant Information Sheet_ PhD Candidates Interview.docx • RS-DFID Consent form_ACBI PhD students_Template.doc Data are available under the terms of the Creative Commons Zero “No rights reserved” data waiver (CC0 1.0 Public domain dedication).

## Software availability

NVivo and SPSS software are only available on licence. Alternative open-source software is available here.
