# Supplementary material for: How an international research programme can contribute to improvements in the research environment: the perspective of doctoral students in sub-Saharan Africa
Source: F1000Res. 2024 Jun 24;13:238. Originally published 2024 Mar 28. [Version 2] doi: 10.12688/f1000research.144883.2 (PMC11165298; doi:10.12688/f1000research.144883.2)
Supplement: Peer review history

# Reviewer report: Reviewer response for version 2

**DOI:** 10.5256/f1000research.167927.r294777

**Published:** 2024-06-25

**Authors:** Donald Cole

**Affiliations:** 1 Dalla Lana School of Public Health, University of Toronto, Toronto, ON, Canada

Appreciate your responsiveness to my review, particularly in methods and terms for data.

Is the work clearly and accurately presented and does it cite the current literature?

Yes

If applicable, is the statistical analysis and its interpretation appropriate?

Not applicable

Are all the source data underlying the results available to ensure full reproducibility?

Partly

Is the study design appropriate and is the work technically sound?

Yes

Are the conclusions drawn adequately supported by the results?

Yes

Are sufficient details of methods and analysis provided to allow replication by others?

Partly

Reviewer Expertise:

Historically research capacity strengthening among others, methodologically mixed methods.

I confirm that I have read this submission and believe that I have an appropriate level of expertise to confirm that it is of an acceptable scientific standard.

# Reviewer report: Reviewer response for version 1

**DOI:** 10.5256/f1000research.158739.r263539

**Published:** 2024-06-10

**Authors:** Francesco Obino, Idah Razafindrakoto

**Affiliations:** 1 Global Development Network, New Delhi, Delhi, India; 2 Research and Programs, Global Development Network (Ringgold ID: 193563), New Delhi, Delhi, India

The article presents the results of an assessment of the impact of linking a doctoral track to an internationally-funded research consortium (ACBI) between UK and African higher education institutions. The study was carried out, and the paper was authored, by the Centre for Capacity Research (CCR) at the Liverpool School of Tropical Medicine as part of their role in monitoring and evaluating the capacity-strengthening components of the ACBI program. Specifically, the paper presents and discusses data relating to the experiences of the involved PhD students (38 in total, of which 33 participated in the study).

 The content is well-structured, moving from background and general considerations to a presentation of results, organized by sub-topics, and then to a discussion. The paper uses adequate citations from interviews and basic statistical analysis of survey responses by the PhD students.

 Our main first comment (of two) focuses on the framing of the paper and its contribution. The title is catchy (for those interested in the topic), but it leads the reader to expect a comprehensive assessment of the impact of the program on A) PhD students' 'research environment and progress' and B) 'in sub-Saharan Africa.' We believe that the framing is too ambitious and does not reflect the content of the paper. Ultimately, the paper presents a 'user-side perspective' of a doctoral track integrated into an international research program. The title should reflect this more accurately and reduce the scope from an entire continent to the specific program at stake. The confusion is genuine, and we see no attempt to misguide the reader, but the title over-commits the authors to something much more ambitious than the available evidence.

 Following the first comment, it emerges from the paper that a separate publication and dataset exist on the perspective of the institutions participating in the program. We understand the incentives for producing separate papers, but it would have been very useful to use the two datasets to answer the broader question regarding the strengthening of doctoral tracks, which is one analytical level up from the experience of the PhD students, as the lens moves from the individual to the institutional level of analysis.

 Ultimately, the paper argues that embedding PhD students 'by design' in an international research consortium can have benefits for the quality of doctoral training received, particularly as the consortium can mobilize resources that single institutions cannot. However, the shortfalls emerging from the PhD students' responses point to a number of more systemic obstacles that are proper to a) doctoral programs (i.e., uneasiness with supervision as a technology of capacity building when the personal match is not right; the bureaucratic nature of higher education institutions, etc.) and b) universities, particularly universities in many African countries (lack of resources, lack of career advisory services, etc.). The risk of this analysis is to implicitly reiterate the 'blanket' narrative about the 'weak' research and research training environment at a continental scale and to miss the critical nuances that, on such a small sample of people and institutions, could lead to opportunities and levers of change.

 It would be very useful to correlate the experience of the students, based on their responses, to their own aspirations and personal trajectories, and to specific realities of their institutions. Ultimately, the paper describes the benefits of being part of an 'elite' PhD cohort, with access to more resources and opportunities for mobility and supervision compared to others and other doctoral tracks that remain undefined and are not described. The strongest asset of the paper is also its Achilles' heel: the idea that an international program is a variable in strengthening a process of research capacity building (such as a doctoral track) in isolation from the research systems from which and in which the students emerge. A mention of these broader 'systemic' aspects and a critical assessment of the normative bias of the program the paper describes towards the benefits of international consortia could help stir some debate on the paper itself.

Is the work clearly and accurately presented and does it cite the current literature?

Yes

If applicable, is the statistical analysis and its interpretation appropriate?

I cannot comment. A qualified statistician is required.

Are all the source data underlying the results available to ensure full reproducibility?

No source data required

Is the study design appropriate and is the work technically sound?

Yes

Are the conclusions drawn adequately supported by the results?

Yes

Are sufficient details of methods and analysis provided to allow replication by others?

Yes

Reviewer Expertise:

research capacity building, social sciences, international funding, ethics of North-South research collaboration

We confirm that we have read this submission and believe that we have an appropriate level of expertise to confirm that it is of an acceptable scientific standard.

# Response

**Published:** 2024-06-13

**Authors:** Susie Crossman

**Affiliations:** Liverpool School of Tropical Medicine, UK

**1. The paper presents a 'user-side perspective' of a doctoral track integrated into an international research program. The title should reflect this more accurately and reduce the scope from an entire continent to the specific program at stake.**

 >>The title has been adjusted to address the reviewers’ concerns

**2. It emerges from the paper that a separate publication and dataset exist**

 >>This refers to the baseline study, please refer to El Hajj T, Gregorius S, Pulford J, et al.: Strengthening capacity for natural sciences research: A qualitative assessment to identify good practices, capacity gaps and investment priorities in African research institutions. *PLoS One.* 2020;15(1):e0228261. 31978119 10.1371/journal.pone.0228261 PMC6980527

**3.…..analysis is to implicitly reiterate the 'blanket' narrative about the 'weak' research and research training environment at a continental scale and to miss the critical nuances that, on such a small sample of people and institutions, could lead to opportunities and levers of change.**

 >>In the discussion we have replaced ‘likely to’ with ‘could’ to reduce the emphasis on the wider applicability of our findings. We hope the actions we have summarised in box 2 are potential ‘levers of change’ that can lead to opportunities to further enhance the research environment for doctoral students everywhere, not just in sub-Saharan Africa.

**4. the paper describes the benefits of being part of an 'elite' PhD cohort, with access to more resources and opportunities for mobility and supervision compared to others…….**

 >>We have added a statement in the discussion to highlight that these were ‘elite’ students.

**5.…..an international program is a variable in strengthening a process of research capacity building (such as a doctoral track) in isolation from the research systems from which and in which the students emerge. A mention of these broader 'systemic' aspects and a critical assessment of the normative bias of the program the paper describes towards the benefits of international consortia could help stir some debate on the paper itself.**

 >>We have emphasized in the discussion that it is important for such international consortia to discover and build on the strengths of their partner institutions, to avoid undermining their systems and to align their efforts with the vision, strategy and needs of partner institutions. 

# Reviewer report: Reviewer response for version 1

**DOI:** 10.5256/f1000research.158739.r276503

**Published:** 2024-06-06

**Authors:** Donald Cole

**Affiliations:** 1 Dalla Lana School of Public Health, University of Toronto, Toronto, ON, Canada

Great to see this work based on interviews with doctoral students, and relevant doctoral studies literature. 

 Abstract provides a good summary of the article.

 Methods seem to start in the Ethics section, not sure why. The mixed methods design would appear to be overlapping based on the timelines identified. Reference to a typology of mixed methods designs would be helpful e.g. Cresswell and Plano's text.(Creswell et al., 2017)(Ref-3)

 More information could be provided on the sources for questionnaire items i.e. prior questionnaires in the literature, conceptual framework of the parent study, etc. Reference to the availability of the questionnaire, and the interview guide in the extended data instruments and consent should be noted in the Methods section.  

 Sources of interview questions are more fully set out.

 Was analysis sequential i.e. questionnaires first as completed first, then interview data? or was it integrated as per the theme description? Currently interview analysis and themes are described prior to questionnaire analysis and results, though participant characteristics described jointly.

 Please clarify each of these so Methods can be rated yes vs partly.

 Findings include some well known but some very exciting newer findings like solidarity among doctoral students in the networking section. 

 Box 1 also helpful as a very positive statement on consortia impact.

 Discussion includes some unnecessary repetition of methods. The links with literature are good, though some on other consortia experience in Africa would be worthwhile e.g. (Adedokun et al.,2014)(Ref-1) or (Ruhweza Katahoire A et l.,2023) (ref-2)

 The very recommendation focused discussion is important for the application of the study, including Box 2. Similarly some good further research questions.

 Among limitations, your note in findings that some interviewees asked that material be "off the record" is an indication of constraints that they felt. What proportion of interviewees indicated this?

 In the conclusions, I would clarify that these were natural science students, as sciences include social and health sciences as well.

 The terms "underlying data" and " extended data" are new to me. It would seem that the latter is really research instruments, data collection and ethics related. The former would seem appropriate for the interview transcripts.  Not clear from the questionnaire data. Can you clarify?

Is the work clearly and accurately presented and does it cite the current literature?

Yes

If applicable, is the statistical analysis and its interpretation appropriate?

Not applicable

Are all the source data underlying the results available to ensure full reproducibility?

Partly

Is the study design appropriate and is the work technically sound?

Yes

Are the conclusions drawn adequately supported by the results?

Yes

Are sufficient details of methods and analysis provided to allow replication by others?

Partly

Reviewer Expertise:

Historically research capacity strengthening among others, methodologically mixed methods.

I confirm that I have read this submission and believe that I have an appropriate level of expertise to confirm that it is of an acceptable scientific standard, however I have significant reservations, as outlined above.

## References

[1] : Sharing perspectives and experiences of doctoral fellows in the first cohort of Consortium for Advanced Research Training in Africa: 2011-2014. *Glob Health Action* .2014;7: 10.3402/gha.v7.25127 25127 10.3402/gha.v7.25127 25280739 PMC4185087

[2] : Transformed through the CARTA experience: changes reported by CARTA fellows about their PhD journey. *Glob Health Action* .2023;16(1) : 10.1080/16549716.2023.2272392 2272392 10.1080/16549716.2023.2272392 37942510 PMC10653687

[3] : Designing and Conducting Mixed Methods Research. *https://us.sagepub.com/en-us/nam/designing-and-conducting-mixed-methods-research/book241842* .2017;

# Response

**Published:** 2024-06-13

**Authors:** Susie Crossman

**Affiliations:** Liverpool School of Tropical Medicine, UK

**1. Methods seem to start in the Ethics section, not sure why. The mixed methods design would appear to be overlapping based on the timelines identified. Reference to a typology of mixed methods designs would be helpful e.g. Cresswell and Plano's text.(Creswell et al., 2017)**

**Was analysis sequential i.e. questionnaires first as completed first, then interview data? or was it integrated as per the theme description? Currently interview analysis and themes are described prior to questionnaire analysis and results, though participant characteristics described jointly.**

 >> the ethics section has been moved to the end of the methods

 >> the timelines for conducting and analysing data from the different methods have been clarified, Briefly, the majority of the baseline questionnaires were completed in May 2018. A few students were unable to complete them online because of difficulties in accessing the form and were therefore given the opportunity to fill them in either offline (via email) or in-person prior to their face to face interviews which were held in either July/ August 2018 or December 2018.

 >> the description of, and rationale for, the mixed methods approach has now been expanded and supported by the helpful Cresswell reference [thank you]

**2. More information could be provided on the sources for questionnaire items i.e. prior questionnaires in the literature, conceptual framework of the parent study, etc. Reference to the availability of the questionnaire, and the interview guide in the extended data instruments and consent should be noted in the Methods section.  Sources of interview questions are more fully set out.**

 >> more details about the sources for the questionnaire and interview items have been provided

 >> the link to the ‘extended data’ additional documents (questionnaire, interview guide, consent) has been inserted in the methods and reference to ‘supplementary file’ deleted.

**3. Please clarify each of these so Methods can be rated yes vs partly.**

 >> see responses above

**4. Discussion includes some unnecessary repetition of methods. The links with literature are good, though some on other consortia experience in Africa would be worthwhile e.g. (Adedokun et al.,2014) or (Ruhweza Katahoire A et l.,2023)**

 >> repetition of methods has been removed from the discussion

 >> additional references relevant to research fellows’ programmes and experiences in Africa have been added [thank you for these]

**5. Among limitations, your note in findings that some interviewees asked that material be "off the record" is an indication of constraints that they felt. What proportion of interviewees indicated this?**

 >> More information on this has been provided in the results section. Only one student did not want their interview recorded: instead running notes were taken during the interview. Three others asked for the recording to be turned off temporarily; this included two who did not want to share personal health information. Three did not request the recording to be stopped but at the end of the interview they asked for specific pieces of information not be to disclosed. Reasons for not recording/disclosing related to concerns about sharing their honest opinion on relationship (power) dynamics with supervisors or colleagues. Nevertheless, the vast majority of interview responses were recorded as the participants trusted that the information would be handled sensitively and anonymously. We have not gone into this level of detail in the text but have adjusted it to reflect that virtually all of the information provided during interviews was recorded. 

**6. In the conclusions, I would clarify that these were natural science students, as sciences include social and health sciences as well.**

 >> this has been clarified

**7. The terms "underlying data" and " extended data" are new to me. It would seem that the latter is really research instruments, data collection and ethics related. The former would seem appropriate for the interview transcripts.  Not clear from the questionnaire data. Can you clarify?**

 >> these are not our terms but are those used by the F1000 journal. Their guidelines state “ *we no longer host supplementary material. Instead, we ask that these be deposited in an approved repository and included in the article’s Data Availability section as “Extended data”*.” In the methods section we have clarified the types of information that are provided as extended data (see response to #2).

# Reviewer report: Reviewer response for version 1

**DOI:** 10.5256/f1000research.158739.r263540

**Published:** 2024-04-30

**Authors:** Julie Reeves

**Affiliations:** 1 University of Southampton, Southampton, England, UK

This mixed method study provides an important insight into the perceptions and experiences of doctoral researchers in international consortia, and continues the research into the ACBI programme. The paper makes a critical addition to our understanding of international consortia and the benefits they are able to bring to doctoral researchers, and their institutions.  We do not know enough about the experiences of researchers in international research partnerships - and this paper makes a very welcome contribution to the field.  One that will be useful to other international research partnerships with doctoral and early career researchers.  It is particularly notable that the voices and perspectives of the African PhD students in the consortia provide the central focus of this research and the paper.  

 The suggestions the authors make towards mitigating the challenges doctoral researchers face and their findings, are especially useful in encouraging consortia to be mindful of the impact they can have in leveraging resources and expertise to support individuals and institutions.  .

 The interview transcripts have not been made available as anonymity cannot be maintained if they were open source (i.e. No - the source data are not available).  However, the interview questions and the questionnaire are available and make it possible for similar studies to be undertaken by other international research consortia. 

 The authors uniquely enquired into the perceptions of doctoral researchers in the consortium.  The interview questions and the questionnaire used to corroborate the interview data, were comprehensive and will have yielded a rich range of data. The study design is consistent and builds on a baseline assessment and an established evidence informed benchrmark approach. The ACBI was a substantial programme and the authors have been able to draw on 35 of 38 doctoral researchers for interview purposes.  The authors make excellent use of the qualitative data and quotations to illustrate their key points. This enables important points about the interviewees' experiences to unfold in their own words.  The paper has a comprehensive structure and is well written. 

 In view of the above, there is a need for some more detail in a few places and to help situate the reader on occasion.  For example, the 'Characteristics of the PhD students' indicates the gender makeup of respondents, but the paper does not indicate whether or not there were any areas of significant gender difference. 

 There is a good summary of the field of study, the nature of the institutions, the stage of study and language in this section, yet the reviewer wondered if it was possible to offer any overall information about the countries in which the respondents are located without compromising anonymity.  As 18 countries were involved in the consortia, were there any notable regional differences? 

 It would be useful to have an indication of the common complaints, with percentages, among the interviewees to follow the demographic/characteristics information. An overall view (perhaps in a table against the key themes) would help to set the scene for the remainder of the paper and the detailed quotations. 

 There are a few areas where an example would assist the reader and add to the paper.  For instance, under the section 'Institutions' research infrastructure and facilities',  an example to illustrate what is meant by 'inadequate research infrastructure and facilities' (is this lab equipment or research management offices, or both?) would help the reader to appreciate the comment that follows. Where some students reported delay due to power outages or those students undertaking computational research who were frustrated by software- are the authors able to say how many students were affected?        

 A minor point - the Introduction paragraph says 'The UK spend on strengthening research capacity in LMIC is vast'.  'Vast' is a strong word and one would want to know how this spend compares with internal research funding or other countries' expenditure, to qualify the term.

 An excellent study and enjoyable paper that will  stimulate reflection on international research partnerships and the experiences of the researchers within them.     

    

Is the work clearly and accurately presented and does it cite the current literature?

Yes

If applicable, is the statistical analysis and its interpretation appropriate?

I cannot comment. A qualified statistician is required.

Are all the source data underlying the results available to ensure full reproducibility?

No

Is the study design appropriate and is the work technically sound?

Yes

Are the conclusions drawn adequately supported by the results?

Yes

Are sufficient details of methods and analysis provided to allow replication by others?

Yes

Reviewer Expertise:

Researcher development, doctoral supervision, research leadership and research capacity strengthening.

I confirm that I have read this submission and believe that I have an appropriate level of expertise to confirm that it is of an acceptable scientific standard.
